# Prevalence, Awareness, Treatment, and Control of Diabetes Among 0.98 Million Patients With Stroke/TIA in China: Insights From a Nationwide Cohort Study

**DOI:** 10.1111/1753-0407.70059

**Published:** 2025-03-02

**Authors:** Siqi Chen, Gulbahram Yalkun, Hongqiu Gu, Xin Yang, Chunjuan Wang, Xingquan Zhao, Yilong Wang, Liping Liu, Xia Meng, Yong Jiang, Hao Li, Yongjun Wang, Zixiao Li, Jue Liu, Donghua Mi

**Affiliations:** ^1^ Department of Neurology, Beijing Tiantan Hospital Capital Medical University Beijing China; ^2^ Department of Neurology Second Affiliated Hospital of Xinjiang Medical University Urumqi China; ^3^ China National Clinical Research Center for Neurological Diseases Beijing China; ^4^ Advanced Innovation Center for Human Brain Protection Capital Medical University Beijing China; ^5^ Research Unit of Artificial Intelligence in Cerebrovascular Disease Chinese Academy of Medical Sciences Beijing China; ^6^ Department of Epidemiology and Biostatistics, School of Public Health Peking University Beijing China

**Keywords:** China, diabetes, epidemiology, prevalence, stroke

## Abstract

**Background:**

A comprehensive epidemiological investigation of the coexistence between diabetes and stroke/TIA in China is urged.

**Methods:**

Data from the Chinese Stroke Center Alliance program, a nationwide multi‐center registry study, were used to detect the prevalence, awareness, treatment, and control of diabetes among stroke/TIA. The distribution of diagnosed and undiagnosed diabetes and prediabetes among stroke/TIA patients was investigated, the medical care around diabetes and their respective risk predictors were analyzed, and the association of all above diabetes characteristics with in‐hospital death was evaluated using multi‐variable Cox regression models.

**Results:**

Of 980 625 patients included, 308 426 (31.5%) had prediabetes, while 365 052 (37.2%) had diabetes, with nearly a third of them undiagnosed (112 969, 30.9%). Of residual aware diabetic patients, 59.0% were treated, with 27.3% controlled. Compared to Han ethnicity, Zhuang ethnicity had a lower prevalence of diabetes (37.3% vs. 35.1%) but were less aware (69.4% vs. 56.5%), treated (59.4% vs. 47.8%), and controlled (27.4% vs. 26.0%). Patients with prediabetes, diagnosed, and undiagnosed diabetes had increasingly higher risks of in‐hospital death (adjusted HR [95% CI]: 1.47 [1.35–1.60]; 2.15 [1.97–2.34]; 4.20 [3.87–4.56], all *p* < 0.001). Unaware and untreated diabetes were independently associated with in‐hospital death (adjusted HR [95% CI]: 1.99 [1.85–2.14]; 2.84 [2.63–3.07, both *p* < 0.001]). Compared with controlled diabetes, those with uncontrolled diabetes had a lower risk of in‐hospital death (adjusted HR [95% CI]: 0.77[0.68–0.88], *p* < 0.001).

**Conclusions:**

The findings indicate that over two‐thirds of stroke/TIA patients are exposed to diabetes in China, causing higher in‐hospital mortality, which should be screened and intervened early.

**Summary**

- Over two‐thirds of stroke/TIA patients are exposed to diabetes and a lack of timely intervention against hyperglycemia in China.
- Stroke/TIA patients with diabetes had an increasingly higher risk of in‐hospital death.

## 1 Introduction

Imposing tremendous health and economic burdens, diabetes and stroke are two of the most common chronic conditions globally [1, 2]. It was estimated that over half a billion people are living with diabetes mellitus in 2021, and the number was projected to increase by 46% in 2045 [1]. Nonetheless, this increasing burden is being further compounded by an aging population and the worldwide epidemic of obesity [3]. Diabetes was reported to double the risk of acute cerebral vascular disease and worsen its outcomes [2, 4, 5, 6]. Although the two medical conditions share several similarities in pathophysiology and frequently coexist [4], a comprehensive epidemiological investigation is sorely lacking, especially in China.

Stroke remains one of the leading causes of death and dependency in China: it was reported to account for 1.57 million deaths in 2018 [7]. In recent decades, China has gradually developed several national registries [8, 9, 10] to comprehensively overview stroke characteristics and clinical practice, and to provide timely evidence for scientific research. Globally, diabetes has been investigated in a wide range of stroke studies [11, 12], but few of them reported diabetes status (diagnosed, undiagnosed, and prediabetes), and medical care around diabetes among this special population.

Given limited evidence regarding full‐scale characteristics of diabetes among patients with acute cerebral vascular diseases, this study used data from 0.98 million stroke/TIA patients enrolled in the Chinese Stroke Center Alliance (CSCA, 2015–2019) to investigate: (1) prevalence, clinical characteristics, spatiotemporal distribution, and social‐economic status of stroke/TIA patients with diagnosed diabetes, undiagnosed diabetes, and prediabetes across mainland China; (2) prevalence, awareness, treatment, and control of total diabetes among stroke/TIA patients, and their respective risk predictors; (3) association of diabetes with in‐hospital death by diabetic status (diagnosed/undiagnosed diabetes, prediabetes) and by awareness, treatment, and control of diabetes.

## 2 Methods

### 2.1 Study Design and Data Resource

As a multi‐center, hospital‐based nationwide registry study, the CSCA enrolled 1 006 798 patients from 1476 hospitals diagnosed with acute ischemic stroke, intracerebral hemorrhage, subarachnoid hemorrhage, or TIA between August 1, 2015 and July 31, 2019. Inclusion and Exclusion criteria of CSCA were presented in Table S1. Detailed rationale and design of the program have been reported elsewhere [13]. Briefly, patients with stroke/TIA confirmed by head CT/MRI within 7 days of symptom onset and aged > 18 years were enrolled in this program. Data were collected through an internet‐based tool (Medicine Innovation Research Center, Beijing, China), including patient demographics; medical and medication histories; hospital presentation, diagnosis, and treatment; hospital complications, outcomes, and fasting blood sample assessment. Data quality was optimized by setting predefined logic features, range checks, and user alerts to identify a potentially invalid format or value entries. Only in‐hospital data were recorded, and patients after discharge were not followed. All participating hospitals in the CSCA were allowed to collect data without requiring individual patient informed consent under the common rule and were approved a waiver of authorization and exemption from their institutional review board.

### 2.2 Study Population

**FIGURE 1:**
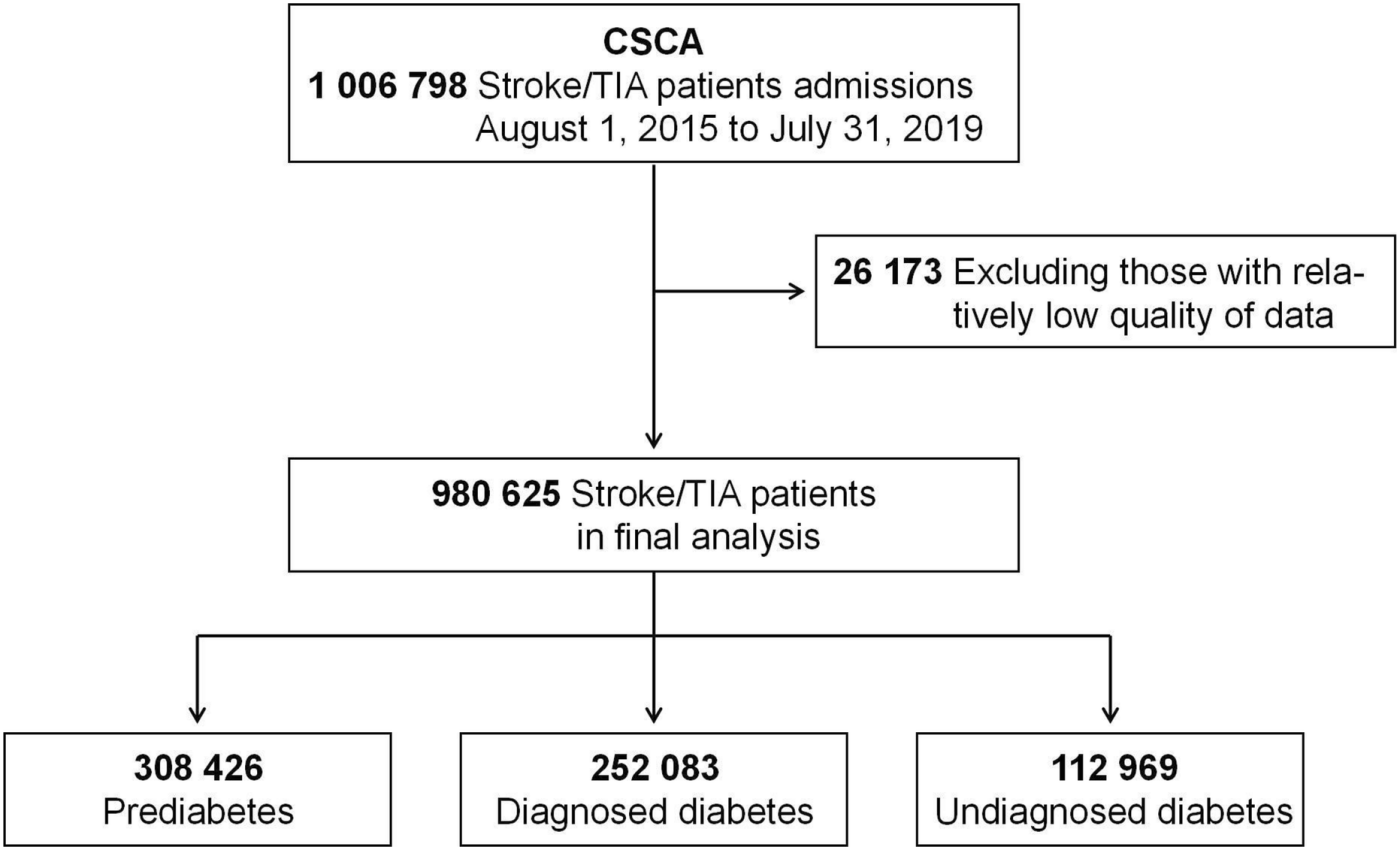
Flow chart of the study.

We included all stroke (acute ischemic stroke, intracerebral hemorrhage, and subarachnoid hemorrhage) or TIA patients enrolled between January 1, 2016 and July 31, 2019. Patients recruited during the year of 2015 (between August 1 and December 31, *N* = 26 173 [2.6%]) were not included in our analysis because of low enrollment rate in this period, and we managed to exclude the possibility that lack of experience in program management at the beginning might generate relatively low quality of data. Therefore, totally 980 625 patients were included in our analysis (Figure 1). Stroke was diagnosed at each hospital by local neurologists according to the 1989 World Health Organization criteria [14] and with confirmation of head CT/MRI. TIA was defined as a brief episode of neurologic dysfunction caused by focal brain or retinal ischemia, with clinical symptoms typically lasting less than 1 h, and without evidence of acute infarction [15]. Stroke/TIA were all diagnosed at each hospital by local neurologists.

### 2.3 Prediabetes and Diabetes

Diabetes was ascertained according to diagnostic criteria from the American Diabetes Association [16]. Diagnosed diabetes was physician‐confirmed diabetes extracted from medical charts on previous medical history or discharge diagnosis. Undiagnosed diabetes was defined as among patients without a discharge diagnosis of diabetes, hemoglobin A1C (HbA1C) ≧ 6.5% on admission. Total diabetes includes diagnosed and undiagnosed diabetes. Prediabetes was defined as among patients without a diagnosis of diabetes, HbA1C was between 5.7% and 6.4% on admission.

Prevalence of diabetes was defined as the proportion of total diabetes among all study individuals. Awareness was defined as the proportion of individuals with history of physician‐diagnosed diabetes among all patients with diabetes. Treatment was defined as the proportion of individuals receiving diabetes medications among all patients with diabetes. Control was defined as the proportion of individuals with an HbA1c level of less than 7.0% among patients with diabetes who were taking medication.

### 2.4 Definition of Variables and In‐Hospital Death

Comprehensive investigation was performed to evaluate baseline characteristics of study subjects: (1) patients demographics: age, sex, ethnic group (Notably, the Chinese people are composed of ethnic Han and 55 ethnic minorities, and among the latter, ethnic Zhuang is the largest. Therefore, ethnic groups were divided into Han, Zhuang, and other); (2) spatiotemporal distribution: the location (belonged region of Eastern/Central/Western China) and the enrolled year of patients (2016–2019); (3) social‐economic status by self‐report: household income (yuan/month), health insurance status (urban employee basic medical insurance, urban resident basic medical insurance, new rural cooperative medical scheme, and others), and educational level (primary school or lower, middle school, high school, college or above, and unknown); (4) medical histories extracted from medical charts: hypertension, dyslipidemia, previous TIA, and previous ischemic stroke; (5) others: current diagnosis (stroke subtype or TIA); smoking status (self‐reported: never smoking, previous smokers [stopped smoking for a year], current smokers, and unknown); drinking alcohol (self‐reported); body mass index (calculated as weight in kilogram divided by the square of height in meters [kg/m^2^], including normal [18.5–24.9], underweight [< 18.5], overweight [17, 18, 19, 20, 21], obese [> = 30], and unknown); and grade of hospital where the patient was enrolled (Grade II or III, hospitals of grade I were not included in the CSCA program due to their less experience of clinical practice and scientific research. Hospitals of Grade III represent the highest level in China).

Defined as death due to any cause during hospitalization, in‐hospital death was reported by neurologists at local hospitals and extracted from medical charts.

### 2.5 Statistical Analysis

Continuous variables were presented as mean (standard deviation, SD), and categorical variables as counts (percentage). Prevalence, awareness, treatment, and control of total diabetes were evaluated (presented as rates and 95% confidence interval [95% CI]), and their possible risk predictors were also analyzed via Logistic regression models by adjusting for age, sex, year, region, ethnic group, educational level, household income, health insurance status, smoking, drinking, BMI, medical histories, current diagnosis, and level of hospital. In addition, we reported the prevalence of diagnosed diabetes, undiagnosed diabetes, and prediabetes in the whole 31 provinces of mainland China, respectively. We also compared the difference in prevalence of diabetes between 2016 and 2019 by province to evaluate relevant changes over time. The impact of diabetes on in‐hospital death by diabetic status (no diabetes, diagnosed/undiagnosed diabetes, prediabetes) and by awareness, treatment, and control of diabetes were investigated via multi‐variable Cox regression models by adjusting for age, sex, year, region, ethnic group, educational level, household income, health insurance status, smoking, drinking, BMI, medical histories, current diagnosis, and level of hospital. All statistical analyses were conducted using SAS software version 9.4 (SAS Institute Inc) and used 2‐sided tests with a significance threshold of 0.05.

### 2.6 Role of the Funding Source

The funder had no role in study design, data collection, data analysis, data interpretation, or writing of the report. The corresponding authors had access to all the data in the study and had final responsibility for the decision to submit for publication.

## 3 Results

### 3.1 Prevalence and Characteristics of Diagnosed Diabetes, Undiagnosed Diabetes, and Prediabetes

**FIGURE 2:**
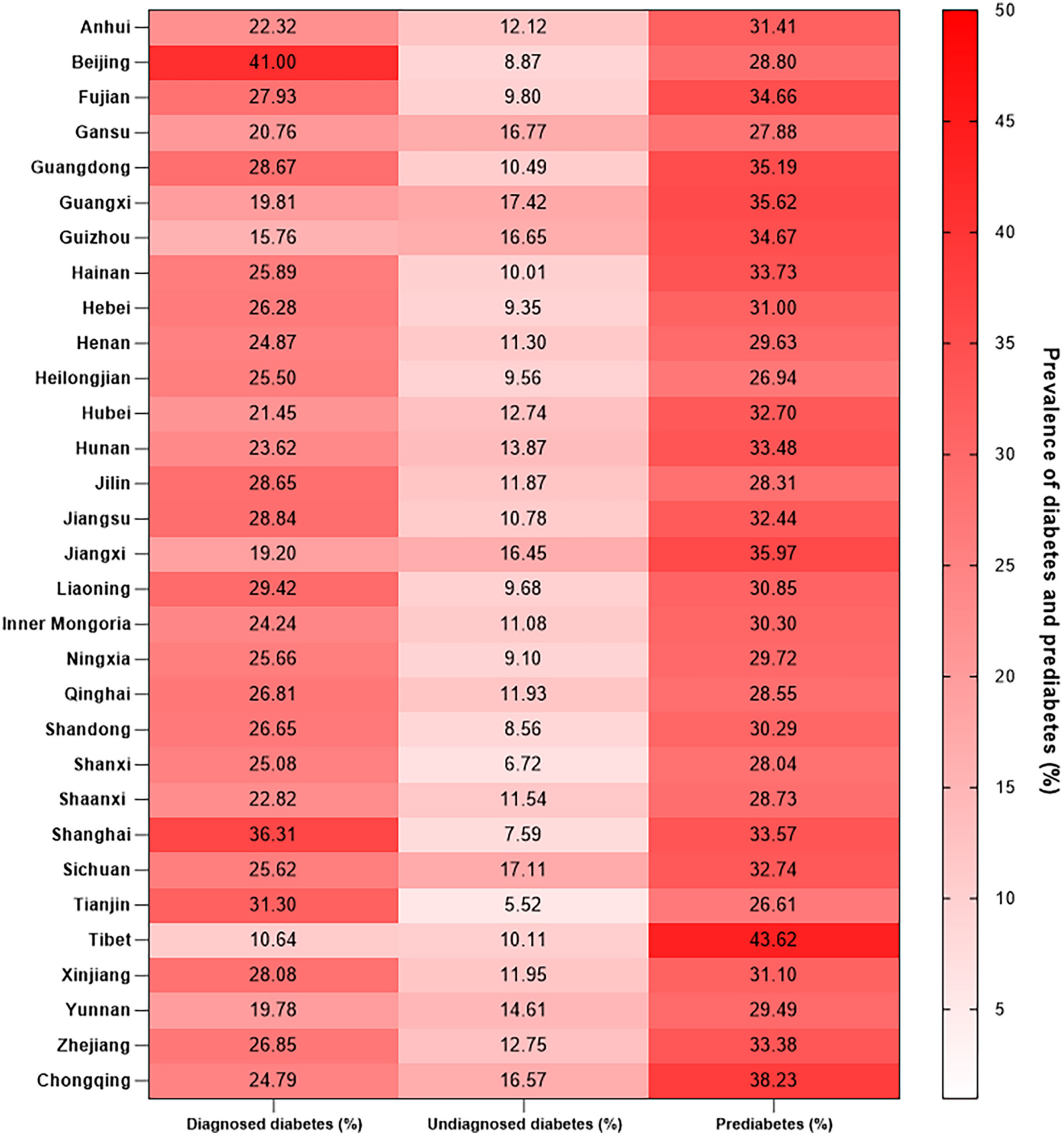
Prevalence of diabetes and prediabetes among 0.98 million patients with stroke/TIA in China by province.

**FIGURE 3:**
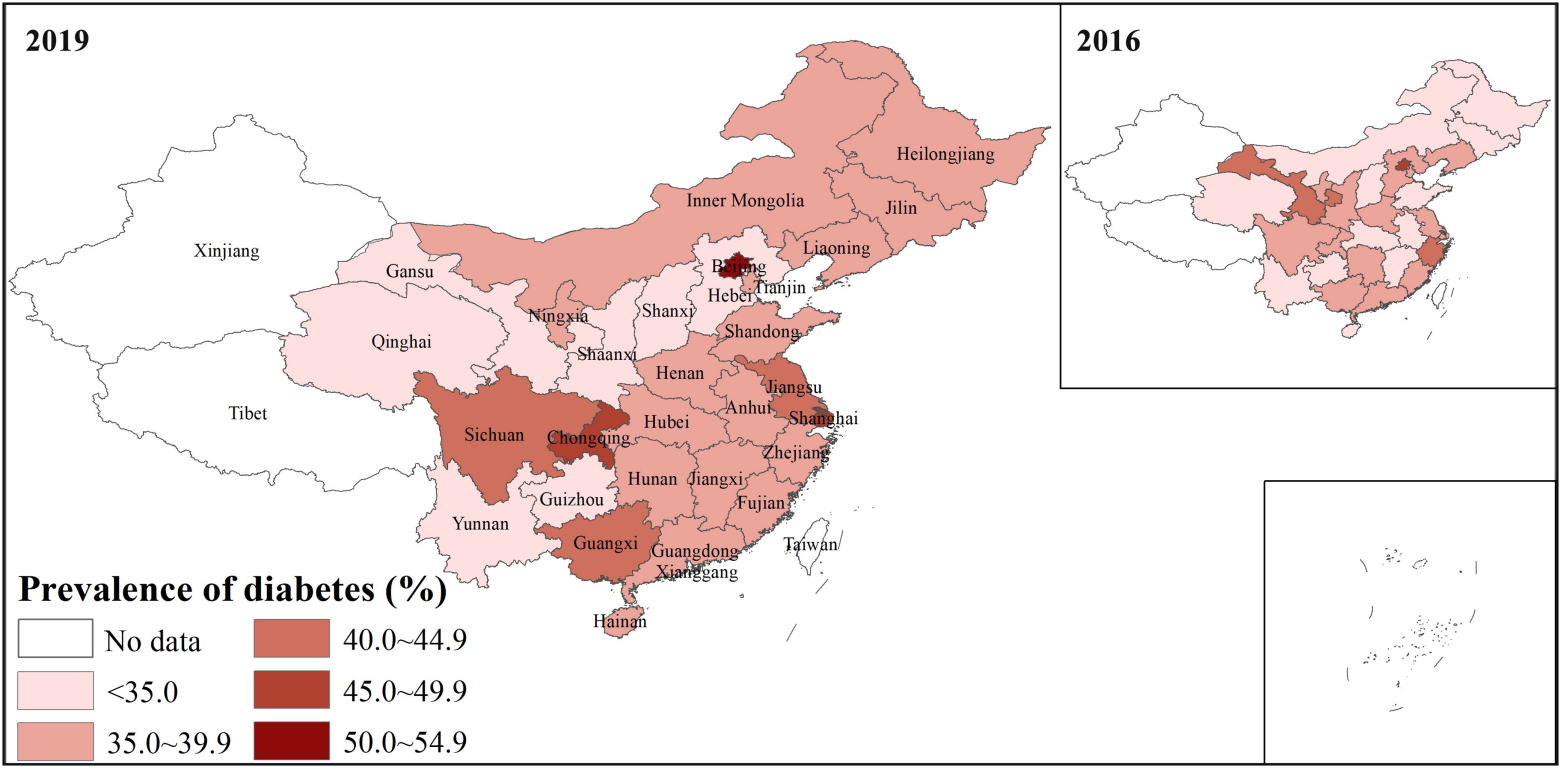
Prevalence of diabetes in China in 2016 and 2019 by province.

Of 980 625 patients included in our analysis: 308 426 had prediabetes (31.5%), and 365 052 had diabetes (37.2%). Among patients with diabetes, 69.0% were diagnosed, while 31.0% were undiagnosed. Baseline characteristics were detailed in Table 1. Figure 2 shows the prevalence of diagnosed/undiagnosed diabetes and prediabetes among stroke/TIA patients in China by province, while Figure 3 evaluates the prevalence of diabetes in 2016 and 2019 by province. It was found that Beijing (41.00%) and Shanghai (36.31%) had the highest rates of diagnosed diabetes and had relatively lower rates of undiagnosed diabetes (8.87% and 7.59%, respectively). Figure 3 demonstrated that the prevalence of diabetes was generally increasing from 2016 to 2019 across mainland China.

**TABLE 1:** Prevalence of diabetes and prediabetes among 0.98 million patients with stroke/TIA in China by basic characteristics.

Characteristics	*N* (%)	Prevalence (%)			*p*
		Prediabetes	Diagnosed diabetes	Undiagnosed diabetes	
Overall	980 625 (100.0)	308 426 (31.5)	252 083 (25.7)	112 969 (11.5)	
Age groups (years)					0.0001
18–29	2948 (0.3)	763 (25.9)	202 (6.9)	316 (10.7)	
30–39	14 795 (1.5)	4424 (29.9)	2137 (14.4)	1508 (10.2)	
40–49	79 253 (8.1)	24 340 (30.7)	16 397 (20.7)	8370 (10.6)	
50–59	194 650 (19.8)	58 814 (30.2)	52 470 (27.0)	20 347 (10.5)	
60–69	302 374 (30.8)	92 708 (30.7)	86 437 (28.6)	32 968 (10.9)	
70–79	251 315 (25.6)	80 423 (32.0)	66 402 (26.4)	30 478 (12.1)	
> = 80	135 290 (13.8)	46 954 (34.7)	28 038 (20.7)	18 982 (14.0)	
Gender					0.0001
Male	607 233 (61.9)	192 314 (31.7)	145 702 (24.0)	67 185 (11.1)	
Female	373 392 (38.1)	116 112 (31.1)	106 381 (28.5)	45 784 (12.3)	
Year					0.0001
2016	220 403 (22.5)	66 872 (30.3)	54 037 (24.5)	25 375 (11.5)	
2017	253 971 (25.9)	79 937 (31.5)	64 647 (25.5)	29 171 (11.5)	
2018	319 014 (32.5)	101 102 (31.7)	83 899 (26.3)	37 025 (11.6)	
2019	187 237 (19.1)	60 515 (32.3)	49 500 (26.4)	21 398 (11.4)	
Region					0.0001
Eastern	438 100 (44.7)	141 422 (32.3)	123 002 (28.1)	43 817 (10.0)	
Central	336 220 (34.3)	100 703 (30.0)	82 298 (24.5)	37 362 (11.1)	
Western	206 305 (21.0)	66 301 (32.1)	46 783 (22.7)	31 790 (15.4)	
Ethnic group					0.0001
Han	947 893 (96.7)	298 009 (31.4)	245 486 (25.9)	107 988 (11.4)	
Zhuang	30 177 (3.1)	9628 (31.9)	5984 (19.8)	4607 (15.3)	
Other	2555 (0.3)	789 (30.9)	613 (24.0)	374 (14.6)	
Education level					0.0001
Primary school or lower	294 386 (30.0)	97 072 (33.0)	69 016 (23.4)	37 197 (12.6)	
Middle school	193 828 (19.8)	60 069 (31.0)	51 194 (26.4)	21 108 (10.9)	
High school	98 018 (10.0)	29 615 (30.2)	28 848 (29.4)	10 153 (10.4)	
College or above	29 870 (3.0)	8850 (29.6)	9046 (30.3)	2881 (9.6)	
Unknown	364 523 (37.2)	112 820 (31.0)	93 979 (25.8)	41 630 (11.4)	
Household income (Yuan/month)					0.0001
< 5000	293 334 (29.9)	93 080 (31.7)	69 246 (23.6)	35 561 (12.1)	
5000–10 000	171 496 (17.5)	54 385 (31.7)	48 307 (28.2)	19 143 (11.2)	
> 10 000	5664 (0.6)	1849 (32.6)	1740 (30.7)	556 (9.8)	
Unknown	510 131 (52.0)	159 112 (31.2)	132 790 (26.0)	57 709 (11.3)	
Health insurance status					0.0001
Uninsured	62 412 (6.4)	20 011 (32.1)	15 014 (24.1)	7528 (12.1)	
NRCMS	419 214 (42.7)	135 913 (32.4)	89 307 (21.3)	50 482 (12.0)	
UEBMI	275 313 (28.1)	81 576 (29.6)	88 259 (32.1)	27 678 (10.1)	
URBMI	182 670 (18.6)	58 540 (32.0)	48 431 (26.5)	21 946 (12.0)	
Other	41 016 (4.2)	12 386 (30.2)	11 072 (27.0)	5335 (13.0)	
Smoking					0.0001
Never smoking	598 898 (61.1)	186 876 (31.2)	162 243 (27.1)	71 456 (11.9)	
Previous smoker	127 794 (13.0)	40 717 (31.9)	32 233 (25.2)	14 872 (11.6)	
Current smoker	226 129 (23.1)	72 246 (31.9)	51 169 (22.6)	22 285 (9.9)	
Unknown	27 804 (2.8)	8587 (30.9)	6438 (23.2)	4356 (15.7)	
Drinking					0.0001
Yes	226 843 (23.1)	72 029 (31.8)	54 275 (23.9)	24 855 (11.0)	
No	727 987 (74.2)	228 422 (31.4)	191 776 (26.3)	84 196 (11.6)	
Unknown	25 795 (2.6)	7975 (30.9)	6032 (23.4)	3918 (15.2)	
BMI group (WHO standard, kg/m^2^)					0.0001
Normal (18.5–24.9)	631 476 (64.4)	202 619 (32.1)	150 101 (23.8)	73 053 (11.6)	
Underweight (< 18.5)	35 262 (3.6)	11 802 (33.5)	5962 (16.9)	4822 (13.7)	
Overweight (25.0–29.9)	260 330 (26.5)	78 957 (30.3)	79 063 (30.4)	27 861 (10.7)	
Obese (> = 30.0)	37 097 (3.8)	10 560 (28.5)	12 944 (34.9)	4901 (13.2)	
Unknown	16 460 (1.7)	4488 (27.3)	4013 (24.4)	2332 (14.2)	
Hypertension					0.0001
Yes	629 858 (64.2)	191 471 (30.4)	186 009 (29.5)	70 729 (11.2)	
No	339 717 (34.6)	113 183 (33.3)	64 132 (18.9)	40 421 (11.9)	
Unknown	11 050 (1.1)	3772 (34.1)	1942 (17.6)	1819 (16.5)	
Dislipdemia					0.0001
Yes	71 558 (7.3)	18 470 (25.8)	30 831 (43.1)	6691 (9.4)	
No	854 314 (87.1)	274 522 (32.1)	202 009 (23.6)	100 763 (11.8)	
Unknown	54 753 (5.6)	15 434 (28.2)	19 243 (35.1)	5515 (10.1)	
Previous TIA					0.0001
Yes	22 839 (2.3)	7020 (30.7)	6032 (26.4)	2566 (11.2)	
No	937 076 (95.6)	295 023 (31.5)	240 280 (25.6)	107 343 (11.5)	
Unknown	20 710 (2.1)	6383 (30.8)	5771 (27.9)	3060 (14.8)	
Previous ischemic stroke					0.0001
Yes	275 964 (28.1)	80 127 (29.0)	85 051 (30.8)	30 107 (10.9)	
No	689 595 (70.3)	223 462 (32.4)	163 389 (23.7)	80 485 (11.7)	
Unknown	15 066 (1.5)	4837 (32.1)	3643 (24.2)	2377 (15.8)	
Current diagnosis					0.0001
Ischemic stroke	817 639 (83.4)	253 123 (31.0)	225 265 (27.6)	85 780 (10.5)	
TIA	63 068 (6.4)	20 409 (32.4)	13 431 (21.3)	5191 (8.2)	
Intracranial hemorrhage	82 543 (8.4)	29 277 (35.5)	11 036 (13.4)	17 424 (21.1)	
SAH	10 818 (1.1)	3808 (35.2)	1064 (9.8)	3399 (31.4)	
Other unallocated stroke	6557 (0.7)	1809 (27.6)	1287 (19.6)	1175 (17.9)	
Level of hospital					0.0001
Secondary hospital	383 263 (39.1)	120 499 (31.4)	88 466 (23.1)	42 708 (11.1)	
Tertiary hospital	597 362 (60.9)	187 927 (31.5)	163 617 (27.4)	70 261 (11.8)	

Abbreviations: NRCMS, new rural cooperative medical scheme; UEBMI, urban employee basic medical insurance; URBMI, urban resident basic medical insurance.

### 3.2 Diabetes Prevalence, Awareness, Treatment, and Control Among Stroke/TIA Patients

Among 365 052 patients with diabetes, 69.1% were aware, and 59.0% were treated. Among those who were treated, only 27.3% were get controlled. Compared to Han ethnicity, Zhuang ethnicity had a lower prevalence of diabetes (37.3% vs. 35.1%) but they were less likely to be aware (69.4% vs. 56.5%), treated (59.4% vs. 47.8%), and controlled (27.4% vs. 26.0%) with diabetes. Generally, those who were of middle age (50–69), female, located in the eastern area, with higher socio‐economic status, overweight/obese, with dyslipidemia/hypertension/previous ischemic stroke, and in tertiary hospitals were more likely to be aware and treated; while diabetes was more likely to be controlled among patients who were female, in the western area, with a higher educational level, lower household income, non‐smokers, with hypertension/previous ischemic stroke, and in secondary hospitals (all *p* < 0.05, Table 2).

**TABLE 2:** Prevalence, awareness, treatment, and control of diabetes and related risk factors among patients with stroke/TIA in China.

Characteristics	Prevalence of diabetes		Awareness of diabetes		Treatment of diabetes		Control of diabetes	
	Prevalence % (95% CI)	Adjusted OR (95% CI)	Prevalence % (95% CI)	Adjusted OR (95% CI)	Prevalence % (95% CI)	Adjusted OR (95% CI)	Prevalence % (95% CI)	Adjusted OR (95% CI)
Overall	37.2 (37.1–37.3)	—	69.1 (68.9–69.2)	—	59.0 (58.9–59.2)	—	27.3 (27.1–27.5)	—
Age groups (years)								
18–29	17.6 (16.2–19.0)	1 (reference group)	39.0 (34.9–43.2)	1 (reference group)	29.5 (25.7–33.6)	1 (reference group)	26.1 (19.7–33.5)	1 (reference group)
30–39	24.6 (23.9–25.3)	1.43 (1.29–1.59)	58.6 (57.0–60.2)	1.91 (1.56–2.35)	46.7 (45.1–48.4)	1.82 (1.47–2.25)	23.1 (21.1–25.1)	0.85 (0.58–1.25)
40–49	31.3 (30.9–31.6)	1.97 (1.79–2.17)	66.2 (65.6–66.8)	2.54 (2.10–3.09)	55.8 (55.2–56.4)	2.53 (2.07–3.09)	24.4 (23.7–25.2)	0.87 (0.60–1.25)
50–59	37.4 (37.2–37.6)	2.54 (2.30–2.80)	72.1 (71.7–72.4)	3.15 (2.60–3.83)	62.3 (61.9–62.6)	3.12 (2.54–3.80)	25.2 (24.8–25.6)	0.88 (0.61–1.27)
60–69	39.5 (39.3–39.7)	2.72 (2.47–2.99)	72.4 (72.1–72.6)	3.01 (2.48–3.65)	62.9 (62.6–63.2)	3.00 (2.46–3.66)	27.1 (26.8–27.5)	0.96 (0.67–1.38)
70–79	38.5 (38.4–38.7)	2.54 (2.30–2.79)	68.5 (68.2–68.8)	2.40 (1.98–2.91)	58.5 (58.2–58.8)	2.40 (1.96–2.93)	29.1 (28.8–29.5)	1.05 (0.73–1.52)
> = 80	34.8 (34.5–35.0)	2.12 (1.93–2.34)	59.6 (59.2–60.1)	1.56 (1.29–1.89)	48.2 (47.7–48.6)	1.53 (1.25–1.87)	29.7 (29.1–30.3)	1.09 (0.76–1.57)
Gender								
Male	35.1 (34.9–35.2)	1 (reference group)	68.4 (68.2–68.6)	1 (reference group)	58.1 (57.9–58.3)	1 (reference group)	27.2 (27.0–27.5)	1 (reference group)
Female	40.8 (40.6–40.9)	1.22 (1.21–1.23)	69.9 (69.7–70.1)	1.18 (1.16–1.20)	60.3 (60.0–60.5)	1.19 (1.17–1.21)	27.5 (27.2–27.8)	0.96 (0.93–0.98)
Year								
2016	36.0 (35.8–36.2)	1 (reference group)	68.0 (67.7–68.4)	1 (reference group)	58.1 (57.8–58.4)	1 (reference group)	28.9 (28.5–29.3)	1 (reference group)
2017	36.9 (36.8–37.1)	1.04 (1.03–1.06)	68.9 (68.6–69.2)	1.02 (1.00–1.04)	59.0 (58.7–59.4)	1.02 (1.00–1.04)	28.6 (28.2–28.9)	0.99 (0.96–1.02)
2018	37.9 (37.7–38.1)	1.09 (1.08–1.11)	69.4 (69.1–69.6)	1.03 (1.01–1.05)	59.2 (58.9–59.5)	1.01 (0.99–1.03)	26.4 (26.1–26.7)	0.89 (0.86–0.91)
2019	37.9 (37.6–38.1)	1.11 (1.09–1.12)	69.8 (69.5–70.2)	1.04 (1.01–1.06)	59.7 (59.3–60.1)	1.02 (1.00–1.04)	25.7 (25.2–26.1)	0.84 (0.81–0.86)
Region								
Eastern	38.1 (37.9–38.2)	1 (reference group)	73.7 (73.5–73.9)	1 (reference group)	63.1 (62.9–63.3)	1 (reference group)	24.7 (24.4–25.0)	1 (reference group)
Central	35.6 (35.4–35.8)	0.93 (0.93–0.94)	68.8 (68.5–69.0)	0.84 (0.82–0.85)	59.0 (58.7–59.3)	0.88 (0.87–0.90)	31.4 (31.0–31.7)	1.35 (1.32–1.38)
Western	38.1 (37.9–38.3)	1.03 (1.02–1.05)	59.5 (59.2–59.9)	0.56 (0.55–0.57)	50.3 (50.0–50.7)	0.63 (0.62–0.64)	27.1 (26.7–27.6)	1.13 (1.10–1.16)
Ethnic group								
Han	37.3 (37.2–37.4)	1 (reference group)	69.4 (69.3–69.6)	1 (reference group)	59.4 (59.2–59.5)	1 (reference group)	27.4 (27.2–27.6)	1 (reference group)
Zhuang	35.1 (34.6–35.6)	0.91 (0.88–0.93)	56.5 (55.6–57.4)	0.80 (0.76–0.83)	47.8 (46.8–48.7)	0.82 (0.79–0.86)	26.0 (24.8–27.2)	0.93 (0.87–0.99)
Other	38.6 (36.8–40.5)	0.98 (0.91–1.07)	62.1 (59.0–65.1)	0.73 (0.63–0.84)	51.6 (48.5–54.7)	0.78 (0.68–0.89)	27.9 (24.1–31.9)	1.04 (0.86–1.27)
Education level								
Primary school or lower	36.1 (35.9–36.3)	1 (reference group)	65.0 (64.7–65.3)	1 (reference group)	55.1 (54.8–55.4)	1 (reference group)	26.9 (26.5–27.2)	1 (reference group)
Middle school	37.3 (37.1–37.5)	1.02 (1.00–1.03)	70.8 (70.5–71.1)	1.12 (1.09–1.14)	61.1 (60.8–61.5)	1.12 (1.09–1.14)	27.3 (26.9–27.7)	1.06 (1.03–1.09)
High school	39.8 (39.5–40.1)	1.04 (1.03–1.06)	74.0 (73.5–74.4)	1.16 (1.13–1.20)	64.2 (63.7–64.7)	1.15 (1.12–1.18)	27.5 (26.9–28.0)	1.10 (1.06–1.14)
College or above	39.9 (39.4–40.5)	1.04 (1.02–1.07)	75.8 (75.1–76.6)	1.29 (1.23–1.36)	66.0 (65.1–66.8)	1.27 (1.21–1.32)	26.7 (25.7–27.7)	1.08 (1.02–1.14)
Unknown	37.2 (37.0–37.4)	0.99 (0.98–1.00)	69.3 (69.1–69.5)	1.04 (1.02–1.06)	58.9 (58.6–59.1)	1.04 (1.02–1.06)	27.7 (27.4–28.0)	1.12 (1.08–1.15)
Household income (Yuan/month)								
< 5000	35.7 (35.6–35.9)	1 (reference group)	66.1 (65.8–66.4)	1 (reference group)	56.5 (56.2–56.8)	1 (reference group)	29.0 (28.6–29.4)	1 (reference group)
5000–10 000	39.3 (39.1–39.6)	1.04 (1.03–1.05)	71.6 (71.3–72.0)	1.04 (1.02–1.06)	62.3 (61.9–62.7)	1.07 (1.05–1.10)	26.1 (25.6–26.5)	0.91 (0.89–0.94)
> 10 000	40.5 (39.3–41.8)	1.06 (1.00–1.12)	75.8 (74.0–77.5)	1.20 (1.08–1.33)	65.6 (63.7–67.6)	1.17 (1.07–1.28)	23.4 (21.3–25.5)	0.80 (0.71–0.91)
Unknown	37.3 (37.2–37.5)	1.04 (1.03–1.05)	69.7 (69.5–69.9)	1.12 (1.10–1.14)	59.2 (59.0–59.4)	1.08 (1.06–1.10)	27.0 (26.7–27.3)	0.90 (0.88–0.93)
Health insurance status								
Uninsured	36.1 (35.7–36.5)	1 (reference group)	66.6 (66.0–67.2)	1 (reference group)	55.5 (54.9–56.2)	1 (reference group)	25.4 (24.6–26.1)	1 (reference group)
NRCMS	33.3 (33.2–33.5)	0.88 (0.87–0.90)	63.9 (63.6–64.1)	0.91 (0.88–0.94)	54.3 (54.1–54.6)	0.96 (0.93–0.99)	27.9 (27.6–28.2)	0.98 (0.94–1.03)
UEBMI	42.1 (41.9–42.3)	1.21 (1.18–1.23)	76.1 (75.9–76.4)	1.51 (1.46–1.56)	66.0 (65.7–66.2)	1.46 (1.42–1.51)	27.7 (27.4–28.0)	1.01 (0.97–1.05)
URBMI	38.5 (38.3–38.8)	1.04 (1.02–1.06)	68.8 (68.5–69.2)	1.10 (1.06–1.14)	58.7 (58.4–59.1)	1.13 (1.09–1.16)	26.0 (25.5–26.4)	0.96 (0.91–1.00)
Other	40.0 (39.5–40.5)	1.12 (1.10–1.15)	67.5 (66.8–68.2)	1.08 (1.04–1.14)	55.9 (55.1–56.6)	1.05 (1.01–1.10)	28.4 (27.5–29.4)	1.07 (1.00–1.13)
Smoking								
Never smoking	39.0 (38.9–39.1)	1 (reference group)	69.4 (69.2–69.6)	1 (reference group)	59.6 (59.4–59.8)	1 (reference group)	27.9 (27.6–28.1)	1 (reference group)
Previous smoker	36.9 (36.6–37.1)	0.95 (0.93–0.96)	68.4 (68.0–68.8)	0.97 (0.95–1.00)	58.9 (58.5–59.4)	1.01 (0.98–1.03)	28.6 (28.1–29.2)	0.97 (0.94–1.01)
Current smoker	32.5 (32.3–32.7)	0.82 (0.81–0.83)	69.7 (69.3–70.0)	0.98 (0.95–1.00)	58.7 (58.3–59.1)	0.96 (0.93–0.98)	24.2 (23.8–24.6)	0.84 (0.81–0.86)
Unknown	38.8 (38.3–39.4)	1.02 (0.98–1.06)	59.6 (58.7–60.6)	0.78 (0.73–0.82)	49.1 (48.2–50.1)	0.81 (0.76–0.86)	31.8 (30.6–33.1)	1.20 (1.10–1.30)
Drinking								
Yes	34.9 (34.7–35.1)	1 (reference group)	68.6 (68.3–68.9)	1 (reference group)	57.7 (57.4–58.1)	1 (reference group)	26.8 (26.4–27.2)	1 (reference group)
No	37.9 (37.8–38.0)	1.00 (0.99–1.01)	69.5 (69.3–69.7)	1.10 (1.07–1.12)	59.7 (59.5–59.9)	1.13 (1.10–1.15)	27.4 (27.2–27.6)	0.92 (0.89–0.94)
Unknown	38.6 (38.0–39.2)	0.96 (0.93–1.00)	60.6 (59.7–61.6)	0.93 (0.87–0.99)	49.8 (48.9–50.8)	0.97 (0.91–1.03)	30.2 (29.0–31.5)	0.95 (0.87–1.04)
BMI group (WHO standard, kg/m^2^)								
Normal (18.5–24.9)	35.3 (35.2–35.5)	1 (reference group)	67.3 (67.1–67.5)	1 (reference group)	57.3 (57.1–57.5)	1 (reference group)	28.0 (27.8–28.3)	1 (reference group)
Underweight (< 18.5)	30.6 (30.1–31.1)	0.81 (0.79–0.83)	55.3 (54.3–56.2)	0.69 (0.66–0.72)	44.8 (43.9–45.8)	0.68 (0.66–0.71)	31.1 (29.8–32.4)	1.16 (1.09–1.23)
Overweight (25.0–29.9)	41.1 (40.9–41.3)	1.24 (1.23–1.25)	73.9 (73.7–74.2)	1.24 (1.22–1.26)	64.0 (63.7–64.2)	1.20 (1.18–1.22)	25.8 (25.5–26.2)	0.89 (0.87–0.91)
Obese (> = 30.0)	48.1 (47.6–48.6)	1.62 (1.58–1.65)	72.5 (71.9–73.2)	1.13 (1.09–1.17)	61.8 (61.1–62.5)	1.07 (1.03–1.10)	24.6 (23.8–25.4)	0.84 (0.80–0.88)
Unknown	38.5 (37.8–39.3)	1.18 (1.14–1.22)	63.2 (62.1–64.4)	0.98 (0.93–1.04)	51.2 (50.0–52.4)	0.90 (0.85–0.95)	34.8 (33.1–36.4)	1.32 (1.23–1.43)
Hypertension								
Yes	40.8 (40.6–40.9)	1 (reference group)	72.5 (72.3–72.6)	1 (reference group)	62.1 (61.9–62.3)	1 (reference group)	28.2 (28.0–28.4)	1 (reference group)
No	30.8 (30.6–30.9)	0.73 (0.72–0.73)	61.3 (61.0–61.6)	0.65 (0.63–0.66)	52.1 (51.8–52.4)	0.72 (0.71–0.73)	24.9 (24.6–25.3)	0.85 (0.83–0.87)
Unknown	34.0 (33.2–34.9)	0.68 (0.65–0.71)	51.6 (50.0–53.2)	0.45 (0.42–0.49)	40.1 (38.5–41.6)	0.50 (0.46–0.53)	22.0 (20.0–24.2)	0.72 (0.64–0.82)
Dislipdemia								
Yes	52.4 (52.1–52.8)	1 (reference group)	82.2 (81.8–82.6)	1 (reference group)	72.9 (72.5–73.4)	1 (reference group)	25.9 (25.4–26.4)	1 (reference group)
No	35.4 (35.3–35.5)	0.56 (0.56–0.57)	66.7 (66.6–66.9)	0.51 (0.50–0.53)	56.6 (56.4–56.8)	0.56 (0.55–0.58)	27.9 (27.6–28.1)	1.17 (1.14–1.21)
Unknown	45.2 (44.8–45.6)	0.85 (0.83–0.88)	77.7 (77.2–78.2)	1.12 (1.07–1.18)	67.5 (66.9–68.0)	1.12 (1.07–1.16)	24.4 (23.7–25.0)	0.91 (0.87–0.96)
Previous TIA								
Yes	37.6 (37.0–38.3)	1 (reference group)	70.2 (69.2–71.1)	1 (reference group)	58.8 (57.8–59.9)	1 (reference group)	30.6 (29.3–31.9)	1 (reference group)
No	37.1 (37.0–37.2)	0.99 (0.96–1.01)	69.1 (69.0–69.3)	1.26 (1.19–1.32)	59.2 (59.0–59.3)	1.30 (1.24–1.36)	27.2 (27.0–27.4)	0.98 (0.92–1.04)
Unknown	42.6 (42.0–43.3)	1.01 (0.96–1.06)	65.3 (64.4–66.3)	0.97 (0.89–1.05)	52.8 (51.8–53.9)	0.98 (0.91–1.07)	28.0 (26.7–29.3)	1.05 (0.94–1.17)
Previous ischemic stroke								
Yes	41.7 (41.5–41.9)	1 (reference group)	73.9 (73.6–74.1)	1 (reference group)	64.7 (64.4–65.0)	1 (reference group)	29.3 (29.0–29.7)	1 (reference group)
No	35.4 (35.3–35.5)	0.87 (0.87–0.88)	67.0 (66.8–67.2)	0.92 (0.90–0.93)	56.6 (56.4–56.8)	0.87 (0.86–0.89)	26.2 (26.0–26.5)	0.87 (0.85–0.89)
Unknown	40.0 (39.2–40.7)	0.79 (0.75–0.83)	60.5 (59.3–61.7)	0.67 (0.61–0.72)	46.8 (45.5–48.0)	0.59 (0.55–0.64)	27.9 (26.3–29.6)	1.00 (0.89–1.11)
Current diagnosis								
Ischemic stroke	38.0 (37.9–38.1)	1 (reference group)	72.4 (72.3–72.6)	1 (reference group)	62.2 (62.1–62.4)	1 (reference group)	26.4 (26.2–26.6)	1 (reference group)
TIA	29.5 (29.2–29.9)	0.68 (0.67–0.69)	72.1 (71.5–72.8)	0.99 (0.95–1.02)	61.2 (60.5–61.9)	0.96 (0.93–1.00)	34.9 (34.0–35.7)	1.46 (1.40–1.52)
Intractanial hemorrhage	34.5 (34.2–34.8)	0.93 (0.92–0.95)	38.8 (38.2–39.3)	0.25 (0.24–0.25)	30.3 (29.8–30.9)	0.28 (0.27–0.28)	35.6 (34.6–36.6)	1.50 (1.43–1.57)
SAH	41.3 (40.3–42.2)	1.30 (1.25–1.36)	23.8 (22.6–25.1)	0.13 (0.12–0.14)	16.2 (15.1–17.3)	0.13 (0.12–0.14)	39.8 (36.3–43.4)	1.83 (1.57–2.12)
Other unallocated stroke	37.5 (36.4–38.7)	1.05 (1.00–1.10)	52.3 (50.3–54.2)	0.48 (0.45–0.53)	43.5 (41.5–45.4)	0.54 (0.50–0.59)	38.5 (35.6–41.4)	1.68 (1.48–1.90)
Level of hospital								
Secondary hospital	34.2 (34.1–34.4)	1 (reference group)	67.4 (67.2–67.7)	1 (reference group)	58.0 (57.7–58.2)	1 (reference group)	29.5 (29.2–29.9)	1 (reference group)
Tertiary hospital	39.2 (39.0–39.3)	1.18 (1.16–1.19)	70.0 (69.8–70.1)	1.06 (1.04–1.08)	59.6 (59.4–59.8)	1.02 (1.01–1.04)	26.1 (25.9–26.4)	0.85 (0.83–0.87)

*Note:* Adjusted for age, sex, year, region, ethnic group, educational level, household income, health insurance status, smoking, drinking, BMI, medical history, current diagnosis, level of hospital in the multivariable logistic regression models.

Abbreviations: NRCMS, new rural cooperative medical scheme; OR, odds ratio; UEBMI, urban employee basic medical insurance; URBMI, urban resident basic medical insurance.

### 3.3 Diabetes and In‐Hospital Death Among Stroke/TIA Patients

In the multivariable Cox regression model, compared with those without diabetes, patients with prediabetes (adjusted HR 1.47 [95% CI: 1.35–1.60]), diagnosed (adjusted HR 2.15 [95% CI: 1.97–2.34]) and undiagnosed diabetes (adjusted HR 4.20 [95% CI: 3.87–4.56]) had increasingly higher risks of in‐hospital death (all *p* < 0.001, Table 3). Those who were unaware (adjusted HR 1.99 [95% CI: 1.85–2.14]) and untreated (adjusted HR 2.84 [95% CI: 2.63–3.07]) were more likely to experience in‐hospital death (*p* < 0.001). However, patients with uncontrolled diabetes had a lower risk of in‐hospital death (Adjusted HR 0.77 [0.68–0.88], *p* < 0.001).

**TABLE 3:** Association between diabetes and the risk of in‐hospital death among patients with stroke/TIA in the Cox regression models.

	In‐hospital death (%, 95% CI)	Crude HR (95% CI)	P	Adjusted HR (95% CI)	*p*
Total	0.6 (0.6–0.6)				
Status of diabetes					
No diabetes	0.3 (0.3–0.3)				
Prediabetes	0.5 (0.4–0.5)	1.62 (1.49–1.76)	< 0.001	1.47 (1.35–1.60)	< 0.001
Diagnosed diabetes	0.6 (0.6–0.7)	2.08 (1.92–2.26)	< 0.001	2.15 (1.97–2.34)	< 0.001
Undiagnosed diabetes	1.8 (1.7–1.8)	5.71 (5.27–6.19)	< 0.001	4.20 (3.87–4.56)	< 0.001
Awareness of diabetes					
Yes	0.6 (0.6–0.7)				
No	1.8 (1.7–1.8)	2.78 (2.60–2.97)	< 0.001	1.99 (1.85–2.14)	< 0.001
Treatment of diabetes					
Yes	0.5 (0.4–0.5)				
No	1.7 (1.6–1.8)	3.75 (3.48–4.04)	< 0.001	2.84 (2.63–3.07)	< 0.001
Control of diabetes					
Yes	0.6 (0.6–0.7)				
No	0.4 (0.4–0.4)	0.66 (0.58–0.75)	< 0.001	0.77 (0.68–0.88)	< 0.001

*Note:* Adjusted for age, sex, year, region, ethnic group, educational level, household income, health insurance status, smoking, drinking, BMI, medical history, current diagnosis, level of hospital in the multivariable Cox regression models.

Abbreviation: HR, hazard ratio.

## 4 Discussion

To our knowledge, this is the first study investigating diabetes status and medical care among nearly 1 million patients with stroke/TIA in China. Among stroke/TIA patients, we found the prediabetes and undiagnosed diabetes were quite prevalent across mainland China; nearly a third of patients were not aware and not treated. Patients with prediabetes, diagnosed diabetes, and undiagnosed diabetes had increasingly higher risk of in‐hospital death; unaware and untreated diabetes conferred a nearly 2‐ and 3‐fold excess risk for in‐hospital death, respectively, but controlled diabetes was an independent risk predictor of in‐hospital death. Our study demonstrated the unsatisfactory medical care around diabetes across mainland China and the demand for an optimal target of glucose control among stroke/TIA patients.

According to nationally representative data from 2015 to 2017 in China, Li and colleagues [22] reported that the prevalence of diabetes among the general population was 12.8%, which is around a third of diabetes proportion among stroke/TIA patients in our study (37.2%). The high rate of missed diagnosed diabetes indicates poor clinical management of hyperglycemia among stroke/neurological physicians in China, demonstrating the demand to strengthen the training of glucose management among these non‐endocrinology doctors. Additionally, diabetes was a well‐known risk factor for acute cerebral vascular diseases, and a considerably large proportion of stroke/TIA patients had diabetes: it was found that 20.3% of ischemic stroke patients in Scotland [11], and 29.6% of ischemic stroke patients in US [12], and 50.0% of stroke patients in India [23] had diabetes; a meta‐analysis (based on Ovid MEDLINE and EMBASE, between 2004 and 2017) including 359 783 individuals found that the prevalence of diabetes among stroke patients was 28% [24]. Rates of diabetes among stroke patients vary greatly, which might be partly caused by different diagnostic methods, ethnic backgrounds, and geographic locations.

Moreover, awareness of diabetes among the general population varies widely (40%–90%) [22, 25, 26], while limited data reported that it among stroke/TIA patients was around 88% [27, 28]. Our study demonstrated that 69.1% of diabetes patients were aware: patients who were < 50 or > =70, male, located in the western area, with lower socio‐economic status, underweight, with fewer medical histories, and in secondary hospitals were less likely to be aware, indicating that enhancing glucose screening among this population with stroke/TIA is necessary. Enhancing public awareness of diabetes and their own status of glucose metabolism is of critical importance because increasing awareness might be translated into improvements in treatment and control.

Similar to our findings, rates of hypoglycemic treatment and control among patients with diabetes were reported to be 49%–88% and 20%–52% [17, 22, 25, 29]. Although many studies [17, 18, 19] set the glucose control target as HbA1c concentration less than 7.0%, a growing body of studies has found that intensive glucose control may not improve patients' cerebral vascular outcomes, but rather increase the risk of hypoglycemic events [20, 21]. Our study found that stroke/TIA patients with controlled diabetes on admission had a higher risk of in‐hospital death, demonstrating the target of HbA1c concentration less than 7.0% may somehow be harmful to this special population. Actually, American and Chinese guidelines have already recommended less stringent glucose control target [30, 31] for those who are older, with comorbidities or cardiovascular complications. Hence, further investigation for the optimal target of diabetes control among stroke/TIA patients is warranted, and a more individualized target of glucose control is preferable.

Our study showed that compared with non‐diabetic patients, patients with diagnosed diabetes had a 1‐fold higher risk, and undiagnosed diabetes had a 4‐fold higher risk of death during hospitalization, indicating one of the key issues to relieve the stroke burden is to enhance medical care around diabetes. Moreover, comparison of diabetic distribution among stroke/TIA patients in each province of mainland China by using the latest data will provide potent evidence for local health agencies in policy making. In addition, stroke/TIA coexists with diabetes and is commonly seen globally, representing a large amount of population and a non‐negligible health burden. Vast amounts of clinical trials concentrated on primary prevention of cerebral vascular diseases among those with diabetes or other vascular risk factors, however, secondary prevention among those stroke survivors, for whom more evidence was needed to improve clinical outcomes, was poorly studied. Therefore, we appeal for more scientific research among stroke/TIA patients who tend to have more comorbidities, like diabetes, and more unfavorable outcomes.

There are some limitations. Firstly, the CSCA program only included Grade II or Grade III hospitals, which are located in urban areas and represent a superior medical level in China. However, rural areas might be facing more severe challenges around medical care of diabetes [32] and cerebral vascular diseases [33] owing to disadvantages in health education and socio‐economic status. Therefore, our findings are more representative of diabetes care among stroke/TIA patients in urban areas of mainland China. Secondly, undiagnosed diabetes and prediabetes were ascertained according to HbA1C level on admission, without testing of 2‐h oral glucose tolerance, which might cause an underestimation of diabetes prevalence. Thirdly, repeat testing was not carried out given the nature of the observational and retrospective study design, and we categorized diabetes and prediabetes only according to 1 test on admission, which might cause disparities between our results and clinical practice.

## 5 Conclusions

Among stroke/TIA patients, the prevalence of diabetes is increasing from 2016 to 2019 across mainland China; prediabetes and undiagnosed diabetes are commonly seen; nearly a third of patients were not aware and not treated, and all of the above demonstrated the unsatisfactory medical care around diabetes. Controlled diabetes was an independent risk predictor of in‐hospital death, indicating that the optimal glucose control target, especially among those with high cerebral vascular risk, should be further investigated. The findings indicate that diabetes is an important and neglected public health problem among stroke/TIA patients in China. Diabetes should be screened and intervened timely among patients with stroke/TIA.

## Author Contributions

Conception: S.C., G.Y., Z.L., J.L., and D.M. Acquisition: S.C., G.Y., C.W., X.Z., Y.W., L.L., H.L., Y.W., Z.L., J.L., and D.M. Analysis: H.G., X.Y., X.M., Y.J., and J.L. Interpretation: S.C., G.Y., H.G., Z.L., J.L., and D.M. Manuscript drafting: S.C., G.Y., H.G., X.Y., C.W., X.Z., Y.W., L.L., X.M., Y.J., and H.L. Manuscript reviewing: Y.W., Z.L., J.L., and D.M.

## Conflicts of Interest

The authors declare no conflicts of interest.

## Supporting information


Table S1.

## Data Availability

All data used for this analysis are available under reasonable request to the corresponding authors.
