# Supplementary material for: Prevalence, Awareness, Treatment, and Control of Diabetes Among 0.98 Million Patients With Stroke/TIA in China: Insights From a Nationwide Cohort Study
Source: J Diabetes. 2025 Mar 2;17(3):e70059. doi: 10.1111/1753-0407.70059 (PMC11872597; doi:10.1111/1753-0407.70059)
Supplement: Supplementary file 1 — Table S1. [file JDB-17-e70059-s001.docx]

**Table I. Inclusion and Exclusion Criteria of the CSCA.**

| Inclusion criteria | 1. aged 18 years or older |
| --- | --- |
|  | 1. have a primary diagnosis of acute stroke/TIA confirmed by head CT or MRI, including acute ischemic stroke, TIA, intracerebral hemorrhage or subarachnoid hemorrhage |
|  | 1. are within 7 days of symptom onset |
|  | 1. are admitted either directly to wards or through the emergency department. |
| Exclusion criteria | Patients with cerebral venous sinus thrombosis or non-cerebrovascular diseases were excluded |
